# FDA greenlights momelotinib (Ojjaara) as the exclusive treatment for myelofibrosis patients battling anemia

**DOI:** 10.1097/MS9.0000000000002690

**Published:** 2024-10-23

**Authors:** Memuna Jehanzeb, Urooj Iqbal, Neha Farhat, Radeyah Waseem, Maryam Shahzad, Mohammed Mahmmoud Fadelallah Eljack

**Affiliations:** a Ayub Medical College, Abbottabad, Pakistan; b Dow University of Health Sciences, Karachi, Pakistan; c Karachi Medical and Dental College, Karachi, Pakistan; d Faculty of Medicine and Health Sciences, University of Bakht Alruda, Ad Duwaym, Sudan

Myelofibrosis (MF) is a clonal hematologic malignancy with progressive bone marrow fibrosis culminating in its failure. Clinical manifestations include nonspecific constitutional symptoms, splenomegaly due to extramedullary hematopoiesis, and anemia. Anemia is the result of an interplay of multiple factors: ineffective erythropoiesis as a result of the replacement of hematopoietic tissue with fibrosis, enlarged spleen trapping red blood cells, and the presence of proinflammatory cytokines that hinder iron metabolism by increasing hepcidin levels. The bleak prognosis following the incidence of anemia makes it the hallmark of treatment. The currently available therapy is recombinant human erythropoietin, which is only effective in half of the patients with decreased serum erythropoietin levels and lasts rarely more than a year^1^. Immunomodulatory agents, such as lenalidomide or pomalidomide, have also been explored to improve anemia; however, their toxicity undermined their benefit^2^. Danazol, a man-made androgen with reduced potency, is also associated with unimpressive improvement of anemia^3^. To tackle this pressing therapeutic gap, Momelotinib (ojjaara), a novel selective inhibitor of JAK1 and JAK2, which also antagonizes activin receptor type 1 (ACVR1) involved in enhancing iron homeostasis and erythropoiesis, has been approved by FDA on 15th September 2023 as the exclusive treatment for myelofibrosis patients battling anemia^4^.

The SIMPLIFY-1 trial conducted on 432 patients compared the reduction in spleen volume in patients receiving either momelotinib or ruxolitinib at 24 weeks. Whilst the primary outcome was not significantly different between the two groups, momelotinib was associated with reduced transfusion-dependence^5^. The SIMPLIFY-2 trial conducted on 156 patients reported similar results^6^. In a phase II study, momelotinib’s unique inhibition of ACVR1/ALK2, crucial in iron homeostasis, lowers hepcidin, thus enhancing blood iron availability for erythropoiesis^7^. Essentially, momelotinib stands out as a disease-modifying treatment, distinct from traditional approaches like transfusions or erythropoiesis-stimulating agents, addressing anemia symptoms while modifying the underlying disease processes.

Despite being a breakthrough in the treatment of Myelofibrosis, Momelotinib has a diverse side-effects profile, with the most commonly reported ones being thrombocytopenia, hemorrhage, fatigue, dizziness, diarrhea, neutropenia, and nausea. Serious (including fatal) infections (e.g. bacterial and viral, including COVID-19) also occurred in 13% of patients treated with OJJAARA^8^. Irreversible peripheral neuropathy seen in a third of patients is another concern that arises with OJJAARA as it may hinder its clinical use. A possible limitation of momelotinib can be inferred from the SIMPLIFY-2 trial, where it failed to produce a robust spleen response in ruxolitinib-intolerant patients^9^. It cannot go unnoticed that Momelotinib is only a palliative therapy; there is no evidence that momelotinib can reverse myelofibrosis or induce cytogenetic or molecular remissions^10^.

Moreover, there is uncertainty regarding the efficacy and safety of this drug in the pediatric population. Available data on its use in pregnant women is insufficient to calculate the risks of major birth defects or miscarriages. However, it may cause embryo-fetal toxicity based on animal reproduction studies^8^.

Momelotinib ameliorates anemia by inhibition of activin receptor type 1 (ACVR1). This therapeutic targeting of ACVR1 provides a promising approach for the treatment of other myeloid neoplasms associated with ineffective erythropoiesis, such as myelodysplastic syndromes with ring sideroblasts or *SF3B1* mutation, especially those with co-expression of *JAK2* mutation and thrombocytosis^11^. The efficacy of this drug has also been assessed in other neoplasms besides myelofibrosis. In a study conducted by Yih-Giun Cherg *et al*., it was found that momelotinib significantly inhibited the growth of viral hepatitis-associated hepatocellular carcinoma cells and reduced the expression of Jak2, indicating its role in reducing tumorigenesis in hepatocellular carcinoma. Furthermore, the combination of momelotinib and sorafenib synergistically suppresses the proliferation of vHCC cells both in vitro and in vivo, effectively reducing the tumor burden^12^. Momelotinib, via JAK2-STAT pathway inhibition, reduces the chemoresistance of temozolomide in glioblastoma^13^.

## Ethical approval

Ethics approval was not required for this editorial.

## Consent

Informed consent was not required for this editorial.

## Source of funding

This research received no specific grant from any funding agency in the public, commercial, or not-for-profit sectors.

## Author contribution

M.J.: conceptualisation; M.J., U.I., and N.F.: writing – original draft; R.W., M.S., M.M.F.E.: writing – review and editing.

## Conflicts of interest disclosure

The authors declare no conflicts of interest.

## Research registration unique identifying number (UIN)

Not applicable.

## Guarantor

Mohammed Mahmmoud Fadelallah Eljack.

## Data availability statement

Data sharing is not applicable to this article as no new data were created or analyzed in this study.

## Provenance and peer review

Not commissioned, externally peer reviewed.

## Assistance with the study

None.
